# Role of Social Capital and Financial Wellbeing in Reaching Successful Entrepreneurial Financial Performance: A Moderated-Mediated Model of Financial Intelligence

**DOI:** 10.3389/fpsyg.2022.843501

**Published:** 2022-03-02

**Authors:** Lei Yao, Da Meng

**Affiliations:** ^1^School of Business, Jilin University, Changchun, China; ^2^School of Marxism, Shenzhen Technology University, Shenzhen, China

**Keywords:** financial wellbeing, financial intelligence, entrepreneurial financial performance, social capital, entrepreneurial intentions

## Abstract

Financial wellbeing is an emerging variable in business psychology that is expected to measure overall financial status and future financial trajectories. Financial intelligence and wellbeing have been key determining factors for the financial performance of entrepreneurs. The present study aimed to examine the crucial financial determinants (financial wellbeing and financial intelligence) and social capital factors for the entrepreneurial intentions and their financial performances among the 326 entrepreneurs in China. The study's findings showed that the key financial indicators and social capital are significantly related to entrepreneurial intentions, which considerably predict the entrepreneurial financial performance. The mediating relationships also reported that entrepreneurial intentions and financial intelligence significantly mediate the relationship among social capital, financial wellbeing, and entrepreneurial financial performance. The present study has highlighted the implications for potential entrepreneurs for improving their financial performance through sustainable social capital, financial wellbeing, and financial intelligence. This study will also help the strategists in screening the individuals registering as entrepreneurs based on their financial intelligence quotient. The present study enriches the literature by offering an integrated model on financial wellbeing and entrepreneurial financial performance.

## Introduction

Network links in businesses and social capital developed from psychological research have significant importance. It has increasingly become a significant aspect in researchers' studies of entrepreneurship, influencing the direction and tendency of network linkages in start-up firms. However, the link between this and the success of entrepreneurship is uncertain. Individual people, groups, and organizations use social networking to access knowledge and insights from the outside, which is critical for the growth and development of both existing and start-up businesses. Because they are “new” and “little,” new enterprises frequently have “new entry faults” and “small size defects,” and hence suffer substantial resource restrictions (Siu and Bao, 2008). Entrepreneurs frequently construct and exploit social networks to access important information and resources, uncover and develop significant possibilities, and nurture key talents to gain a competitive edge and assure the new venture's long-term viability (Sorenson et al., 2008). Network orientation is the inclination or attitude of using the internet to address difficulties in entrepreneurial activity. As previously stated, new initiatives in China's developing economy confront a variety of challenges, including a shortage of resources and financing to acquire those resources (Long et al., 2016). Consequently, they seek social capital partnerships to tackle these challenges, resulting in far more network-oriented entrepreneurship.

According to researchers, companies are entwined in certain social interactions, and their growth is inextricably impacted by these connections (Watson, 2007). When innovators or new firms actively construct and grow network ties for commercial conduct, they have a connection-oriented attitude of the foundation for gaining and retaining a competitive edge (Strobl and Kronenberg, 2016; Mu et al., 2017). According to studies, the social capital theory refers to the presence of social networks wherein people build unique social ties due to their connections with others and the benefits or knowledge that individuals get as a result of these interactions. People's breadth of knowledge, depth, and efficiency are improved by intimate social engagement (Avotra et al., 2021). An organizations' competitive edge will be improved and preserved if they can swiftly notice market developments and respond and act fast in their supply chain management (Qin et al., 2021; Yingfei et al., 2021). Consequently, if we can comprehend the beneficial structural capital of socializing, as well as the main mental system and common trust relationship, we can not only induce one another's all-round knowledge acquisition but also increase the frequency of knowledge exchange, thereby increasing the willingness and ability of organization members to share knowledge (Rasool et al., 2019; Samma et al., 2020; Zhou et al., 2021).

Scholars have looked at the influence of familial, regional, and karma social capital differences on innovative enterprises (Lans et al., 2015). With the advancement of technology and changes in society's economic structure, corporate sector management entrepreneurs' social capital has evolved as well, with more sources and a larger variety. Bonding social capital, which has strong relationships, and bridging social capital, which has weak ties, are two types of social capital (Jensen and Jetten, 2015). Following the financial crises of 2007–2008, the financial crises of 2010 in Europe, and the financial crisis of coronavirus disease 2019 (COVID-19), the global economy has grown more challenging and complex (Huo et al., 2021). Individuals and workers of various companies both benefit from making sound financial decisions. One's financial wellbeing plays a significant part in having a healthy and productive financial life. Everyone's financial wellbeing, whether individual or family, as well as societies and countries, has become a hot concern. The degree of income a person earns is not entirely determined by their financial circumstances. Some people, for example, are content with their little salary and choose to live within their means rather than wait for a higher degree of financial security. On the other hand, people enjoy a high level of financial security (Taft et al., 2013; Sayed et al., 2020; Li et al., 2021).

Before, the study revealed that financial wellbeing involves more than just money, and financial wellness has a wider definition that includes financial wellbeing. The terms “financial wellbeing” vs. “financial wellness” were frequently misconstrued. Financial wellbeing as a phenomenon is still in its infancy and is being studied in a variety of areas (Kempson et al., 2017). The majority of research on financial wellbeing has indeed been done in industrialized nations. The terms “financial capacity,” “financial literacy,” and “financial wellbeing” are frequently confused. The terms “financial wellbeing” and “financial competence” are not interchangeable. Financial wellbeing refers to a person's ability to satisfy his present obligations, feel confident about his financial future, and make decisions that allow him to enjoy life. Across the globe, people are becoming increasingly concerned about their financial wellbeing (Mahendru, 2021). The formulation of entrepreneurship intention and the execution of entrepreneurship behavior are the two stages of the entrepreneurship process. All entrepreneurship begins with forming an entrepreneurial intention, which has a strong predictive value for entrepreneurship behavior. Many researchers are now focusing on entrepreneurial intention, and they have investigated the elements influencing individual entrepreneurship intention from various perspectives. The entrepreneurial intention is undeniably an essential prerequisite for the growth of entrepreneurship. The impact of relevant research findings on our knowledge of the entrepreneurial process is beneficial (Kong et al., 2020). The role of entrepreneurial intention in moderated mediated studies has been reported before (Tsai et al., 2014).

Business intelligence is a type of financial intelligence. It mostly refers to the use of artificial intelligence (AI) technology and a large amount of electronic financial data from a company to create a computer simulation for a traditional financial analysis model in order to obtain an assessment and prognosis report on the company's operations. Deloitte, one of the world's largest four major accountancy firms, partnered with Kira Systems on March 10, 2016, to formally bring AI into finance operations. Financial management has reached a new epoch. Following Deloitte's lead, numerous well-known accounting companies globally implemented AI and offered the financial robotic service. Kingdee debuted a cloud-based financial robot at the 2017 Global Forum of China Management in Shanghai. In 2016, robot process automation (RPA) technology was adopted by more than 10% of enterprises throughout the world (Gou, 2020). With the advent of such intelligent systems, it became easy for moderating relationships of factors such as social capital and financial wellbeing on entrepreneurial performance and achieving success.

However, there is a lack of study interest on the social capital of entrepreneurs and its impact on entrepreneurial performance or the success in the field of corporate sector management of China. The research conducted by Xie et al. (2021) focused on social capital and its components such as bonding and bridging in relationship to entrepreneurial performance in the agricultural context, leaving a research gap for future researchers to find the impact of other factors along with social capital on entrepreneurial success and performance in other fields as well. Moreover, the research also found moderating roles of operational competency and opportunity recognition toward entrepreneurial performance supporting a moderation of opportunity capacity. This indicated the role of testing some other moderators between both variables. Financial intelligence is an emerging field in business management so it provided us an opportunity for testing the moderating mediated role of it in our research model. Developed countries like America, England, Australia, and France are in search of devising and defining the financial wellbeing of the people associated with different businesses and startups. A clear understanding of financial wellbeing is still not found and the literature available also does not have great support for the concept of financial wellbeing (Mahendru, 2021). This poses a huge gap in the field of business management, so the role of financial wellbeing has a great scope to be explored in various contexts of management. The mediating role of entrepreneurial intention has also been studied by Mwiya et al. (2018) in the context of nascent behaviors and self-efficacy. This suggested its mediating role in our context between social capital and entrepreneurial performance in the corporate sector management in China.

Based on these gaps in the research of social capital and financial wellbeing, entrepreneurial financial performance indicating the success of entrepreneurship, and a few questions are raised, and their possible solution is explored through the following objectives: (1) To analyze the impact of social capital on entrepreneurial financial performance in the corporate sector management of China. (2) To address the relationship of financial wellbeing with entrepreneurial financial performance. (3) To investigate the mediating role of entrepreneurial intentions between social capital and entrepreneurial financial performance. (4) To evaluate the moderating mediated effects of financial intelligence between financial wellbeing and entrepreneurial financial performance.

## Review of Literature and Hypotheses Development

Our research was in the direction of evaluating entrepreneurial financial performance with the mediation of entrepreneurial intentions and moderation of financial intelligence. The factors and their interaction were based on two following theories of self-efficacy and the social capital theory of management.

### Self-efficacy Theory

The social learning theory's self-efficacy (SE) terminology refers to a person's belief in their capacity to do a certain activity (Bandura, 1977). Self-efficacy is a significant component in judging the value of a human activity, and research reveals that people who have high self-efficacy for a task are more likely to start and finish it. One of the most important aspects of self-effectiveness is the mastery of the work at hand. Researchers emphasize that self-efficacy is measured in a particular region and measures success expectations in that area. The inclusion of self-efficacy inside the model demonstrates that the birds provide insight into the cognitive process. Various tasks are used to generate and keep commercial intents (Boyd and Vozikis, 1994; Eccles, 1994). The planned construction enterprise is influenced by self-effectiveness. It shows that the students are self-assured in their ability to succeed in business and handle problems (Boyd and Vozikis, 1994). Previous studies might be utilized to relate corporate self-efficiency with business aims. Some people feel that corporate self-effectiveness and commercial purpose are related to each other (Bharanti, 2016). Ajzen's self-efficacy was seen, as well as the closest related to perceived behavior control. Self-efficacy is used to execute managerial behavior in research. Individual perceptions of their abilities to undertake commercial operations are stimulated by the desire to establish a firm (Rakib et al., 2020). According to studies, a direct correlation between business efficiency and entrepreneurial intent has been identified. Self-efficacy is connected to the function of entrepreneurial orientation to entrepreneurialism as a mediator between perceived behavior management and self-efficacy. In another study, perceived behavioral control was employed as a mediator between perceived social standards and older business goals (Tsai et al., 2014). These kinds of relationships studied before for the utilization of self-efficacy theory in different perspectives led to entrepreneurial intention and provided us the opportunity to analyze the mediating role of entrepreneurial intention in our context. So it led to the development of the mediator which was entrepreneurial intention between social capital, financial wellbeing, and entrepreneurial financial performance.

Rogers (1975) Protection motivation theory (PMT) model was created to explain how fear appeals affect health attitudes and behaviors. Fear-inducing communications have a significant influence on behavior selection. Improvements in the perceived level of fear frequently led to increases in the acceptability of the adaptive control action or intention, according to a meta-analysis of studies of fear-arousing messages published between 1953 and 1980 (Sutton and Hallett, 1988). Increases in perceived response efficacy also enhanced the likelihood of choosing the ability to respond. The theory has been used to a varied range of themes, including areas of interest outside health-related difficulties, according to a detailed narrative assessment of the literature and research on theory (Prentice-Dunn et al., 1997). The theory has been expanded to environmental concerns, prevention of accidents, safeguarding others, and political issues in addition to health promotion and illness prevention.

### Social Capital Theory

According to social capital theory, social ties are resources that may help people grow and accumulate human capital. A solid family culture, for example, might encourage scholastic achievement and the acquisition of hugely valuable and valued abilities and credentials. Social capital may be described as any element of a social interaction that provides reproduction advantages in evolutionary terms (Bourdieu, 2008). There are many different definitions, interpretations, and applications of social capital. The popularity of social capital among policymakers, according to David Halpern, stems from the concept's duality, which “has a hard-nosed economic sense while restating the centrality of the social.” The phrase is popular among academics in part because of the vast range of outcomes it may explain. The variety of use for social capital has led to a variety of definitions. Superior management performance, the emergence of entrepreneurial enterprises, the increased performance of functionally varied groups, the value generated from strategic partnerships, and improved supply-chain linkages have all been explained using social capital at various periods. Changes in the relationships among actors produce a resource that actors acquire from certain social systems and then employ to achieve their goals (Moran, 2005; Bhandari and Yasunobu, 2009; Stam et al., 2014).

The existing and perceived resources available throughout a performer's system of relations are referred to as social capital. The underlying assumption is that expenditures in social interactions produce benevolence that may be mobilized to accomplish certain objectives (Nahapiet and Ghoshal, 1998; Adler and Kwon, 2002). As a result, social capital produces benefits by offering privileged access to academic, monetary, and material heritage to well-connected players. Social capital has arisen as a situational supplement to theories concentrating on personal features in the area of entrepreneurship, recognizing that businesspeople are immersed in a social environment that both facilitates and restricts action (Aldrich and Zimmer, 1986). The spike of studies that have examined connections and entrepreneurship consequences at various levels, such as the influence of public capital in the generation of different firms, the effectiveness of organizational strategic priorities, the creativeness of clusters, as well as the transition of organizational fields, reflects the notoriety of network-based theorizing (De Carolis et al., 2009; Lechner et al., 2010; Wijk et al., 2013; Stam et al., 2014). All these bases provided significant insight into the concept of social capital which we utilized in devising our research model in connection to entrepreneurial financial performance.

### Relationship of Social Capital With Entrepreneurial Intentions and Financial Wellbeing

Social psychologists coined the term “social capital” to describe the utilization of broad relational entrenched ties, such as neighborhood, colleagues, employers, and relationships with family and friends, to assist individuals in building social capital and wealth. Social capital is defined by researchers as a long-term interpersonal relationship that serves as a solid basis for being grouped in the following: collaboration and collectivism (Burt, 1997; Jacobs, 2016). The social capital hypothesis emphasizes how long-term interactions may be a valuable component for network members. A combination of the most significant resources for entrepreneurs, including social capital at the individual and society levels, is referred to as social capital. This research focused on the social capital of entrepreneurs. Bourdieu was the first to define social capital (Xie et al., 2021). Social resources are social capital from a functional standpoint. According to some academics, social resources are divided into two categories: individual assets and public resources (Coleman, 1988). According to him, social resources are embedded in a network of personal ties and arise from a person's personal interactions, and social resources are formed only when an individual interacts with other members of society. He developed the social capital hypothesis as a result of this, describing social capital as “the social resources it includes in social network interactions and may generate returns.”

The social capital of entrepreneurs is defined in this study as the numerous networks of interactions and the consequent social resources of entrepreneurs in the process of beginning a firm, based on Lin's research (Lin et al., 2001). Internal networks and the resources they bring with them are known as bonding social capital, have strong relational features; exterior networks and interactions, and the resources contained in them, known as bridging social capital, that have weak relational characteristics (Sajuria et al., 2014; Ceci et al., 2020). The former facilitates entrepreneurs in obtaining information from outside the organization, identifying opportunities, and gaining decision-making advantages through internal interactions, sharing information, and promoting trust among internal members; the latter facilitates entrepreneurs in obtaining information from outside the organization, sharing information, and promoting trust among internal members (Xie et al., 2021). The role of social capital in financial wellbeing has been studied in many contexts before such as Yeo and Lee (2019), Ceci et al. (2020), and Thomas and Gupta (2021). Similarly, the relationship between social capital and entrepreneurial intentions have also been studied before by Ali and Yousuf (2019) and Yeo and Lee (2019) which suggested analyzing the impact of social capital both in the context of corporate sector management in China toward entrepreneurial performance so we devised the following hypothesis.

***H***_**1.**_ *Social capital has a positive effect on financial well-being*.
***H***_**2.**_ *Social capital has a positive effect on entrepreneurial intentions*.

### Relationship of Financial Wellbeing With Entrepreneurial Intentions and Entrepreneurial Intelligence

Financial wellbeing is a subset of wellbeing, which encompasses contentment with all elements of one's life. As people seek financial success, the notion of financial wellbeing has grown in popularity among scholars and policymakers in recent years. Various fields, such as public health, economics, medicine, and psychology, have investigated financial wellbeing (Kasser and Ryan, 1999; Helliwell and Putnam, 2004). Without a clear understanding of its conception and concepts, financial wellbeing has been mistaken for financial wellness, financial health, and financial fitness. Several studies have included financial wellbeing as a key variable of interest without specifying the construct they are attempting to assess (Gutter and Copur, 2011; Gerrans et al., 2013; Heckman et al., 2014; Hsu et al., 2016; Jimenez-Solomon et al., 2016). The sum of an individual's material resources, reported as financial assets and liabilities, is evaluated as an objective measure of financial wellbeing. Objective measurements show unbiased aspects of a person's financial status. To objectively quantify financial wellbeing, many aspects of objective measurements such as demographic factors, socioeconomic level, consumption, and saving behavior have been employed (Blanchflower David and Oswald, 1999; Rutherford and Fox, 2010). Subjective measurements explain contentment with a particular condition of finance based on a perceived comprehension of debt and savings in order to recreate dynamic information about people's financial wellbeing that is impossible to foresee using objective financial wellbeing measures. Subjective factors assist policymakers in examining people's views and reactions to their financial situation (Delafrooz and Paim, 2011; Sorgente and Lanz, 2019). The relationship between financial wellbeing (due to the non-distinctiveness of the concept) and entrepreneurial intentions and entrepreneurial intelligence has not been studied well before. The various relationships of financial wellbeing with entrepreneurship were somehow reported in the past such as Sánchez-García et al. (2018), Wiklund et al. (2019), and Mahdzan et al. (2020). Its standing proves a possible relationship so, we devised the following hypothesis for the evaluation of entrepreneurial performance.

***H***_**3.**_ *Financial wellbeing has a positive effect on entrepreneurial intentions*.
***H***_**4.**_ *Financial wellbeing has a positive effect on financial intelligence*.

### Relationship of Entrepreneurial Intentions on Entrepreneurial Financial Performance and Its Mediating Role

A lot of research has been carried out in defining the role of entrepreneurial intentions on entrepreneurial performance in the past such as Radipere and Ladzani (2014). They evaluated the business performance in connection to the entrepreneurial intentions and found a significant impact of entrepreneurial intentions previously. Similarly, another research was conducted in the past to evaluate the role of entrepreneurial intentions and entrepreneurship on achieving entrepreneurial success (Lee and Kim, 2013). They also found a significant impact of entrepreneurial intentions on entrepreneurial success. In contrast to checking the impact of intention on success or entrepreneurial performance, Park (2017) conducted research on checking the impact of entrepreneurship on entrepreneurial intentions and found significant relationships. Many scholars have studied entrepreneurial purpose as a social phenomenon in the realm of entrepreneurship. Individual entrepreneurial ambition, in particular, has increasingly been recognized as mainstream in new business strategies. Furthermore, there is substantial evidence that, practically, all recent research serves as key models for entrepreneurial literature. As a result, the ensuing beneficial effects on job generation must be investigated. As a result, entrepreneurial ambitions are strongly linked to conduct, attitudes, subjective standards, and perceived behavioral restrictions (Samma et al., 2020). The mediating role of entrepreneurial intentions has also been studied by many researchers in the past such as Mwiya et al. (2018). These studies and literature allowed us to develop a hypothesis as follows.

***H***_**5.**_ *Entrepreneurial intentions lead to entrepreneurial success*.
***H***_**8.**_ *Entrepreneurial intentions mediate the relationship between social capital and entrepreneurial success*.
***H***_**9.**_ *Entrepreneurial intentions mediate the relationship of financial wellbeing and entrepreneurial success*.

### Relationship of Financial Intelligence With Entrepreneurial Performance and Its Moderating Mediated Role

While practitioners and academics have studied and applied the notions of financial intelligence and financial intelligence quotient as they relate to individuals, the operational definition and scope of these concepts have not been sufficiently precise. Although some experts think that financial intelligence might boost business performance, they have not been precisely applied to the company. The current global financial crisis has brought attention to the need for increased financial literacy among decision-makers in order to improve their capacity to make sound financial decisions and improve their wellbeing. As a result, financial education or financial intelligence is important in the decision-making process (Kamil et al., 2014). In the empirical literature of decision-making, the concept of financial intelligence is relatively new. There have been very few empirical and theoretical investigations on the issue. As a result, despite some evidence in the literature, the academic community has not offered adequately rigorous definitions for financial intelligence. Financial intelligence has been conflated with financial literacy and financial knowledge in the literature, and no scientific measure of financial intelligence has been produced (Kamil et al., 2014). As it is a new concept in the era of artificial intelligence discussed in the introduction, it could have a significant impact on achieving successful entrepreneurial performance in the corporate management sector of China. Based on the above arguments, it can be hypothesized that it could have moderating mediated effects between social capital and entrepreneurial financial performance. In these connections, we devised the following hypothesis for the testing.

***H***_**6.**_ *Financial intelligence has a positive effect on entrepreneurial success*.
***H***_**7.**_ *Financial intelligence moderates the relationship of entrepreneurial intentions and entrepreneurial success*.
***H***_**10.**_ *Financial intelligence mediates the relationship of financial wellbeing and entrepreneurial success*.

**Figure 1:**
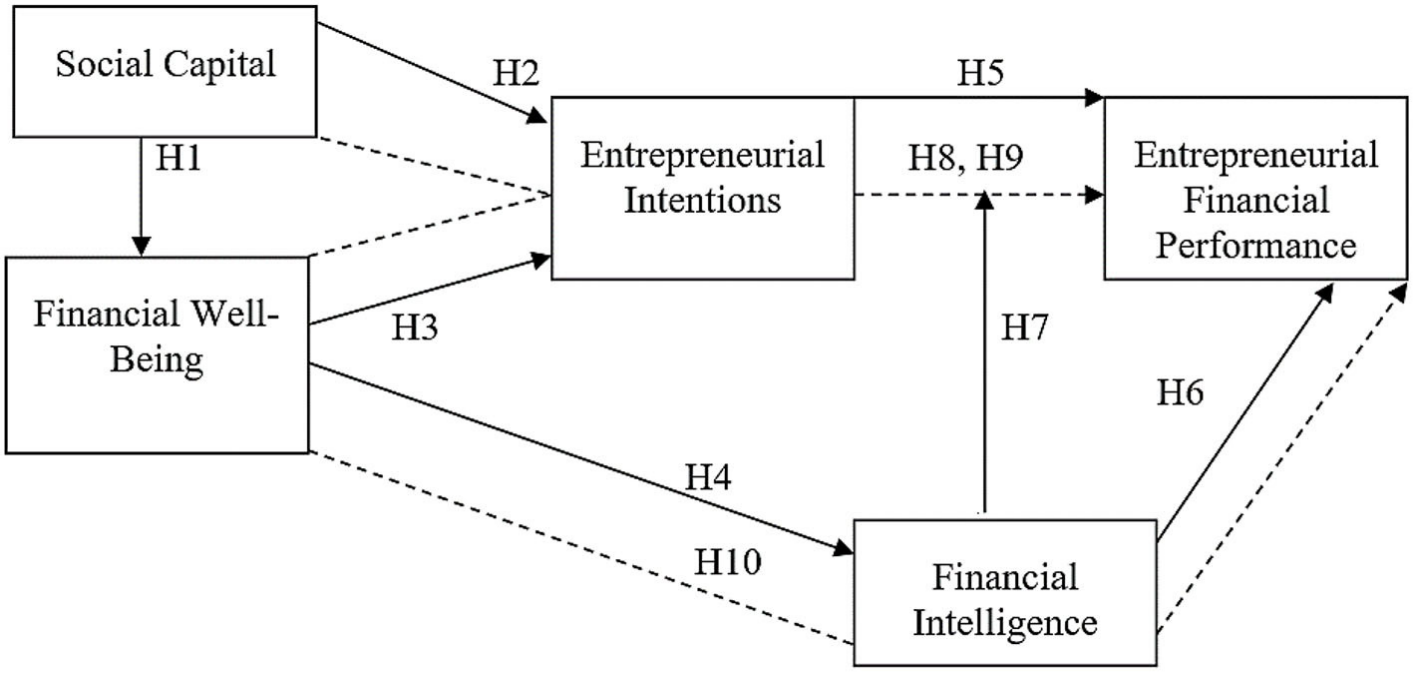
Conceptual model. SC, social capital; FWB, financial well-being; EI, entrepreneurial intentions; FI, financial intelligence; EFp, entrepreneurial financial performance.

This study was based on the following conceptual framework as shown in Figure 1.

## Methodology

In this study, the post-positivist approach to the research philosophy has been adapted since the effects of social capital and financial wellbeing were checked on the other variables of entrepreneurial intentions and entrepreneurial financial performance. In addition, financial intelligence has been proposed to moderate the relationship between entrepreneurial intention and entrepreneurial financial performance. This is a quantitative study with a deductive approach of the research as hypotheses have been proposed and checked empirically later to form the theories. The population taken for the present study is the employees of the corporate sector of China, necessarily involved in entrepreneurial activities. Since it is not possible to take information about all such populations, a non-probability sampling technique has been used. Among those techniques, purposive and snowball sampling techniques have been used (Alias et al., 2020; Adom et al., 2021). This particular population was targeted for the present study because only individuals involved in entrepreneurial activities could generate better results for the study rather than those not involved. Further, employed individuals were targeted because those involved in sole entrepreneurial activities are difficult to approach and get the surveys filled. The data had been collected through the survey method. An earlier consent had been taken if they want to participate in the study and they were ensured that their responses are kept anonymous. The potential agreed participants were informed prior to the actual survey to ensure their availability. The questionnaires were circulated to the participants and they were given the time they needed to fill out the survey. The authors administered all the surveys to avoid any misunderstanding or help needed while answering the questionnaires. For this study, questionnaires were considered as the best option as it completely documents the responses and somewhat indicates the severity of the responses. The data collected through questionnaires were analyzed using the structural equation modeling software Smart-PLS (SmartPLS GmbH, Germany).

### Instrument Development

The data collection instrument used in this study is the questionnaire. There were five variables in total in the study. There is one independent variable, i.e., social capital, one dependent variable of entrepreneurial financial performance, two mediating variables, i.e., financial wellbeing and entrepreneurial intention, and one moderating variable of financial intelligence. This moderating variable of financial intelligence checks if its presence strengthens or weakens the relationship between entrepreneurial intentions and entrepreneurial financial performance. The total number of items in the questionnaire used for this study was 30. The measurement scales used in this study were the most relevant and adapted from those previous studies. The scale for the social capital variable consisted of 7 items and was adapted from Kouvonen et al. (2006). This scale covers all three aspects of social capital including bridging, bonding, and linking. The variable of financial wellbeing contains 8 items addressing the financial happiness and satisfaction of the respondents. It has been adapted from Prawitz et al. (2006). The variable of financial intelligence has been adapted from Michael Collins and Urban (2020), which consists of 5 items addressing scenarios that may be faced by the respondents and how will they cope with those situations regarding their finances. The entrepreneurial intentions have been measured with a 4 item scale that has been previously used by Miralles et al. (2016) in their study. Lastly, the study's dependent variable of the study, i.e., entrepreneurial financial performance, has been measured with a six-item scale previously used by Khan et al. (2021) in the study that revealed significant results.

### Demographics Details

In the first section, the data about the demographics of the respondents were collected. Among the respondents, males and females were found to be equal in numbers. Regarding age, the highest numbers of respondents were from the age 31-above (45.39%), followed by 26–30 categories (34.66%). Regarding education, the highest number of respondents had a master's degree (57.97%), followed by the other category (26.07%) as depicted in Table 1.

**Table 1:** Demographics analysis.

**Demographics**	**Frequency**	**Percentage (%)**
**Gender**		
Male	162	49.70
Female	164	50.30
**Age**		
20–25	65	19.93
26–30	113	34.66
31-above	148	45.39
**Education**		
Bachelors	52	15.95
Masters	189	57.97
Others	85	26.07

*N = 326*.

## Data Analysis and Results

The data analysis of the study was done *via* software commonly used for the structural equation modeling (SEM) analysis, i.e., Smart-PLS ver 3. Through this software, the impact of social capital is checked on the financial wellbeing, and then the simultaneous impact of social capital and financial wellbeing is checked on the entrepreneurial intentions. The mediating effect of entrepreneurial intentions has been checked between the relationship of social capital, financial wellbeing, and entrepreneurial financial performance. Furthermore, the data has been analyzed statistically in two phases, i.e., measurement model and structural model. The measurement model produces the initial results for cleaning the data from spurious items. In the second stage of the structural model, the hypothesis is measured and checked for its significance and final rejection or acceptance.

### Measurement Model

**Figure 2:**
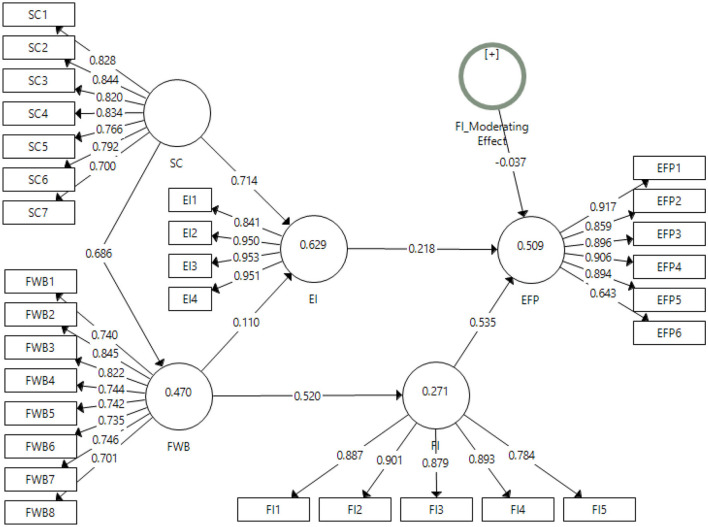
Output of measurement model algorithm. SC, social capital; FWB, financial well-being; EI, entrepreneurial intentions; FI, financial intelligence; EFP, entrepreneurial financial performance.

In this study, the initial screening of the obtained data was done through the results obtained from the measurement model of Smart-PLS. The data have been screened based on the discriminant and convergent validities, reliabilities, r-square values, and average variance extracted. The output algorithm for the measurement model is presented in Figure 2.

The statistics of factor loadings, Cronbach alpha reliability, composite reliability, and average variance extracted (AVE) have been checked for the early screening of the data obtained for each variable of the study. The threshold mentioned in the literature for Cronbach alpha and composite reliability is 0.7 (Nawaz et al., 2019). The values obtained in this study for alpha reliabilities have been well above the threshold reported in the literature. The minimum value for alpha reliability is 0.897, while it is 0.916 for composite reliabilities. Thus, the data is reliable in this study. Moving to the factor loadings, the criteria in the literature mentioned 0.7 as the minimum value, however, a value as low as 0.6 is acceptable (Avotra et al., 2021; Xiaolong et al., 2021). The minimum value in the present study for factor loadings obtained is 0.643 for item EFP6 which is acceptable for an item to fall in that factor. Hence, the item was retained. Furthermore, the threshold for AVE has been 0.5 (Zuo et al., 2018). The minimum value obtained for AVE is 0.578 for the variable financial wellbeing. The present study meets all these criteria for the validities and reliability of the data. The results have been reported in Table 2.

**Table 2:** Factor loadings, reliabilities, and average variance extracted (AVE).

**Variables**	**Factor loadings**		**Cronbach alpha**	**Composite reliability**	**AVE**
**Entrepreneurial financial performance**	EFP1	0.917	**0.925**	**0.943**	**0.736**
	EFP2	0.859			
	EFP3	0.896			
	EFP4	0.906			
	EFP5	0.894			
	EFP6	0.643			
**Entrepreneurial intentions**	EI1	0.841	**0.943**	**0.959**	**0.855**
	EI2	0.950			
	EI3	0.953			
	EI4	0.951			
**Financial intelligence**	FI1	0.887	**0.919**	**0.939**	**0.756**
	FI2	0.901			
	FI3	0.879			
	FI4	0.893			
	FI5	0.784			
**Financial well-being**	FWB1	0.740	**0.897**	**0.916**	**0.578**
	FWB2	0.845			
	FWB3	0.822			
	FWB4	0.744			
	FWB5	0.742			
	FWB6	0.735			
	FWB7	0.746			
	FWB8	0.701			
**Social capital**	SC1	0.828	**0.905**	**0.925**	**0.638**
	SC2	0.844			
	SC3	0.820			
	SC4	0.834			
	SC5	0.766			
	SC6	0.792			
	SC7	0.700			

*N = 326. The bold values indicate significance of the variables*.

The r-square values obtained were significant for the respective dependent variables, explaining a good variance in the model based on each variable. The values obtained for entrepreneurial financial performance R^2^ = 0.509, entrepreneurial intentions R^2^ = 0.629, financial intelligence R^2^ = 0.271, and financial intelligence R^2^ = 0.470. The highest variance explained in this study is by the variable entrepreneurial intention which is 62.9%, followed by entrepreneurial financial performance and financial intelligence. The discriminant validities of the study have been checked through the most commonly used tests of hetero trait mono trait ratio (HTMT) and the Fornell and Larcker Criterion. The results for the HTMT ratio are given in Table 3. The criteria for HTMT results to be significant is that these values should be <0.90 (Franke and Sarstedt, 2019). All the values in the result generated for the HTMT ratio have been found significant.

**Table 3:** Hetero trait mono trait ratio (HTMT) ratio.

	**EFP**	**EI**	**FI**	**FWB**	**SC**
EFP					
EI	0.613				
FI	0.746	0.659			
FWB	0.497	0.619	0.551		
SC	0.647	0.848	0.800	0.733	

*SC, social capital; FWB, financial well-being; EI, entrepreneurial intentions; FI, financial intelligence; EFP, entrepreneurial financial performance*.

Likely, the other statistical test used is Fornell and Larcker criterion. All the values generated for this test are supposed to have the highest value at the top of each corresponding column (Henseler et al., 2015). So, the values for the Fornell and Larcker criterion have been in the same pattern as have been defined. The results for the Fornell and Larcker criterion are given in Table 4.

**Table 4:** Fornell and Larcker criteria.

	**EFP**	**EI**	**FI**	**FWB**	**SC**
EFP	**0.858**				
EI	0.573	**0.925**			
FI	0.686	0.615	**0.870**		
FWB	0.469	0.599	0.520	**0.761**	
SC	0.592	0.789	0.733	0.686	**0.799**

*SC, social capital; FWB, financial well-being; EI, entrepreneurial intentions; FI, financial intelligence; EFP, entrepreneurial financial performance. The bold values indicate significance of the variables*.

### Structural Model

**Figure 3:**
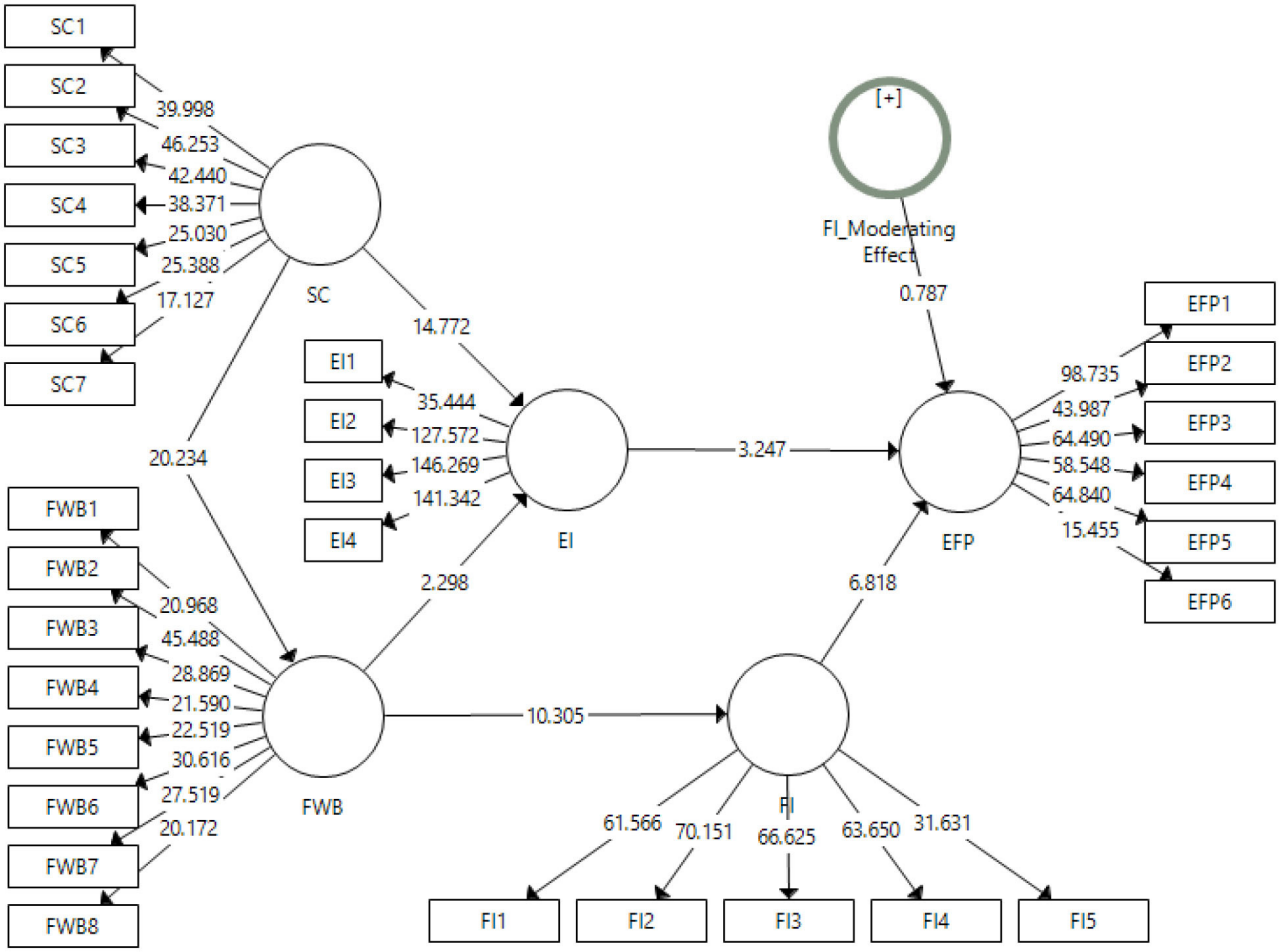
Output of structural model algorithm.

The structural model algorithm has been presented in Figure 3. This output for the structural model shows the significance of the relationships that have been developed in the framework.

The results for the structural model have been shown in Table 5 (direct effects) and Table 6 (indirect effects). These tables elaborate on the results obtained based on the sample means, t-statistics, and *p*-values of the respective hypothesis.

**Table 5:** The direct effects of the variable.

**Paths**	**H**	**O**	**M**	**SD**	**T-statistic**	***P*-value**	**Results**
SC → FWB	H_1_	0.686	0.689	0.034	20.234	0.000^***^	***Accepted***
SC → EI	H_2_	0.714	0.709	0.048	14.772	0.000^***^	***Accepted***
FWB → EI	H_3_	0.110	0.113	0.048	2.298	0.022^*^	***Accepted***
FWB → FI	H_4_	0.520	0.524	0.050	10.305	0.000^***^	***Accepted***
EI → EFP	H_5_	0.218	0.217	0.067	3.247	0.001^**^	***Accepted***
FI → EFP	H_6_	0.535	0.532	0.079	6.818	0.000^***^	***Accepted***
FI_Mod → EFP	H_7_	−0.037	−0.045	0.047	0.787	0.432	Rejected

*** *p < 0.001*;

** *p < 0.005*;

* *p < 0.05; H, Hypothesis; O, Original Sample; M, Sample Mean; SD, Standard Deviation; SC, Social Capital; FWB, Financial Well-being; EI, Entrepreneurial Intentions; FI, Financial intelligence; EFP, Entrepreneurial Financial Performance*.

**Table 6:** The indirect effects of the variable.

**Paths**	**H**	**O**	**M**	**SD**	**T-statistic**	***P*-value**	**Results**
SC → EI → EFP	H_8_	0.156	0.154	0.048	3.226	0.001^**^	***Accepted***
FWB → EI → EFP	H_9_	0.024	0.025	0.014	1.759	0.079	Rejected
FWB → FI → EFP	H_10_	0.279	0.278	0.044	6.276	0.000^***^	***Accepted***

*** *p < 0.001*;

** *p < 0.005; H, Hypothesis; O, Original Sample; M, Sample Mean; SD, Standard Deviation; SC, Social Capital; FWB, Financial Well-being; EI, Entrepreneurial Intentions; FI, Financial intelligence; EFP, Entrepreneurial Financial Performance*.

There are seven hypotheses in this study that report the direct effects of the variables on other variables. Out of these seven hypotheses, six have been accepted except the moderating effect of financial intelligence on entrepreneurial financial performance which is H_7_. In the first hypothesis, social capital has shown a positive and significant effect on the financial wellbeing of the entrepreneurs [β = 68.6%, t-stats = 20.23 (0.000)]. The second hypothesis has been accepted with [β = 71.4%, t-stats = 14.77 (0.000)]. The third and fourth hypothesis for the effects of financial wellbeing on entrepreneurial intentions [β =11%, t-stats = 2.29 (0.022)] and financial intelligence [β =52%, t-stats = 10.30 (0.000)] have also been found significant. Moving to the fifth and sixth hypothesis of the study for the effect of entrepreneurial intention [β = 21.8%, t-stats = 3.24 (0.001)] and financial intelligence [β = 53.5%, t-stats = 6.81 (0.000)] on entrepreneurial financial performance have been approved with a *p* < 0.005.

The indirect effects of the variables for the current study have been mentioned in Table 6. The indirect effects of the variables in this study have been reported in Table 6. There were three hypotheses addressing the indirect effects, i.e., H_8_, H_9_, and H_10_. Out of these three hypotheses, H_9_ has been rejected. The hypothesis H_8_ addressing the mediating role of entrepreneurial intentions among the social capital and entrepreneurial financial performance that has been accepted [β = 15.6%, t-stats = 3.22 (0.001)], while H_10_ has been accepted at *p* < 0.001 [β = 27.9%, t-stats = 6.27].

## Discussion

This study revolved around certain objectives such as analyzing the relationships of social capital on entrepreneurial financial performance, relationships of social capital with entrepreneurial intentions, and financial wellbeing. This study also examined the impact of financial wellbeing on entrepreneurial intentions and entrepreneurial financial performance. The roles of entrepreneurial intentions toward successful entrepreneurial financial performance were also evaluated. Similarly, this study also analyzed the mediating roles of entrepreneurial intentions between social capital and successful entrepreneurial performances. This study also provided insights about the relationships of entrepreneurial intelligence which is a newer concept in the era of artificial intelligence with other certain factors such as entrepreneurial performance and financial wellbeing. The moderating role of entrepreneurial intelligence was also evaluated in this study. This study yielded some interesting results which were in agreement with several previous studies. The first hypothesis was about the role of social capital on financial wellbeing. This hypothesis was accepted, indicating a positive significant role in achieving financial wellbeing. If intentions are there, then financial wellbeing could be achieved in the context of successful entrepreneurial financial performance. The networking would be fruitful in this regard. These confirmed the results of Yeo and Lee (2019).

The results of the second hypothesis indicated the significant relationship between social capital and entrepreneurial intentions. It indicated that networking could result in a huge impact on developing intentions among the entrepreneurs which would lead to successful entrepreneurial performance. These results were in accordance with many researchers in the past (Ali and Yousuf, 2019). The next hypothesis was about the relationship of financial wellbeing with entrepreneurial intentions. The entrepreneurial intentions were also checked for their mediating role between the variables of this study. The direct relation of financial wellbeing with entrepreneurial intentions proved a significant relationship. This indicated the usefulness of financial wellbeing. Psychological wellbeing has been enormously studied before in developing entrepreneurial intentions but our study provided significant contribution in identifying the significant relationship of financial wellbeing with the intentions of the entrepreneurs. Some of the previous researchers also found significant results in the relationship of financial wellbeing with entrepreneurship. Our fourth hypothesis checked the relationship of financial wellbeing with entrepreneurial intelligence. This was also accepted due to the fact that financial wellbeing could lead to mental satisfaction in terms of psychology and would lead toward using financial intelligence in achieving successful entrepreneurial performance. Although the direct relationship of financial wellbeing with entrepreneurial intentions and entrepreneurial intelligence has not been studied before some researchers found the relationship of financial wellbeing with entrepreneurship (Sánchez-García et al., 2018).

The fifth and sixth hypotheses were also accepted indicating the role of entrepreneurial intention and financial intelligence on entrepreneurial financial performance. These two hypotheses indicated the direct role of intentions and intelligence in achieving successful entrepreneurial performance. The intention does not always lead to successful entrepreneurship (Park, 2017), but most of the time, it leads to success in entrepreneurship as found by Lee and Kim (2013). This indicates a stronghold of intentions on achieving success. This is due to the nature of thinking which leads to doing hard work to achieve something targeted. Similarly, financial intelligence could provide insights about predicting the outcomes way ahead in the times of artificial intelligence. These results confirmed the outcomes of some previous researchers (Radipere and Ladzani, 2014). The results of the seventh hypothesis indicated that there is no need for moderation in our model but still, there is a scope of identifying the moderation between social capital directly with entrepreneurial financial performance. This hypothesis was about the moderation between mediator (entrepreneurial intentions) and the entrepreneurial financial performance. It neglected the role of such kind of moderation required when there is a direct relationship significant between entrepreneurial intention and the entrepreneurial financial performance of the corporate sector management.

The eighth and ninth hypotheses evaluated the mediating role of entrepreneurial intention between social capital and entrepreneurial financial performance and the financial wellbeing and the entrepreneurial financial performance. The direct relationships of social capital and financial wellbeing with entrepreneurial financial performance were not studied in our model and the results indicated the positive mediating role of entrepreneurial intention between social capital and entrepreneurial financial performance. This indicated that the mediating role of intentions could boost the relationship of both in a significant way. More attention should be given to entrepreneurial intentions while proposing such a kind of relationship. The mediating role of intentions was proved insignificant between financial wellbeing and entrepreneurial financial performance. It indicated that financial wellbeing is a strong indicator, and it could help in achieving successful entrepreneurial performance without any mediation of entrepreneurial intentions. Although, some other factors could be used in the future for checking the mediating effect between both factors. The mediating roles of entrepreneurial intention were also studied before by Mwiya et al. (2018), and were positively significant.

The mediating role of the moderator of this study, which was financial intelligence, was proved to be significantly mediating the moderated relationship of social capital with entrepreneurial financial performance. The direct relation of financial wellbeing with entrepreneurial financial performance was successfully mediated by financial intelligence leading to the notion that financial intelligence is a strong tool in the era of AI as it provides a handful of insights about achieving success in entrepreneurial financial activities. Financial wellbeing on its own also has a strong influence on entrepreneurial success and that could be enhanced more with the mediation of financial intelligence. Some of the past researchers diligently provided insights for the use of financial intelligence (Gou, 2020). These results could lead to developing a strong nexus between financial wellbeing and entrepreneurial success. This research as a whole would provide a significant contribution to the field of corporate sector management in China.

## Theoretical Contribution

Following are the theoretical contributions of the study. First of all, social capital, financial wellbeing, and financial intelligence have not been studied earlier in a composite framework in the context of the financial performance of entrepreneurs. Therefore, this study adds another direction to the literature on entrepreneurship and financial wellbeing. Secondly, the mainstream intelligence quotient that has been studied is emotional intelligence, however, from the business perspective, financial intelligence needs to be studied to find its contribution to the entrepreneurial behaviors and performances of the entrepreneurs. Thirdly, measuring the role of social capital in financial wellbeing has been really important to understand how the healthy intangible relationships of the entrepreneurs in the form of social capital contribute to the financial wellbeing of entrepreneurs. This study measures financial intelligence and financial wellbeing in the context of making financial decisions that affect the financial performance of entrepreneurs.

## Limitations and Future Research

Along with the theoretical and practical implications of the study, there are a few limitations as well. First, the present study has considered the corporate organizations of China only who are part-time working on their entrepreneurial ventures. Second, a large data set should be used for analysis and a longitudinal study could not be conducted due to lack of time. Thirdly, the data from different geographical regions should be collected and the results should be compared to get an idea if any cultural differences cause any deviation in the results. It is recommended to conduct future studies considering the individuals who are sole entrepreneurs. Secondly, this study should be replicated in developing countries of Asia like Sudan, Sri Lanka, and Indonesia to understand if any difference in the results is found. Third, these relationships should be checked with more financial determinants (like mindful spending, spending behaviors, and financial knowledge) of entrepreneurship and organizational performance to understand the variations in these variables. Furthermore, the moderation effect of other variables like financial understanding, financial literacy should also be checked in future studies.

## Practical Implications

The following are the practical implications of the study. First, this study will be significant for the individuals involved in the entrepreneurial activities that how they should work on enhancing and flourishing their social relationships to grow toward better financial wellbeing which automatically contributes to the financial intelligence that is needed for smart decision making. This whole scenario automatically affects the financial performance of the entrepreneurs. Therefore, understanding and considering the effects of social capital and financial wellbeing will help the individuals who are intending to start up their business venture that how working financially intelligent can positively affect their financial performances in the long run.

## Conclusions

The present study has distinguished the financial wellbeing and financial intelligence of the entrepreneurs as important turning points in their financial performance. The entrepreneurs who have been involved in greater social interactions were found to have better overall financial wellbeing and financial intelligence. The mediation analysis of the study also suggested that financial wellbeing and financial intelligence play an important role for entrepreneurs in their financial performances. This study has also highlighted that social capital and financial wellbeing are vital constituents of entrepreneurial intentions. Further, financial intelligence has also been found as the key determinant of entrepreneurial financial performance. However, the moderation effect of financial wellbeing could not be found to have any significant role in affecting the relationship of entrepreneurial intentions on entrepreneurial financial performance. Therefore, the present study proposes to future entrepreneurs how financially intelligently working toward the goals can benefit them in achieving optimal financial performance.

## Data Availability Statement

The original contributions presented in the study are included in the article/supplementary material, further inquiries can be directed to the corresponding author.

## Ethics Statement

The studies involving human participants were reviewed and approved by Jilin University (JU), China. The patients/participants provided their written informed consent to participate in this study. The study was conducted in accordance with the Declaration of Helsinki.

## Author Contributions

LY conceived and designed the concept and wrote the paper. DM collected the data. All authors read and agreed to the published version of the manuscript.

## Conflict of Interest

The authors declare that the research was conducted in the absence of any commercial or financial relationships that could be construed as a potential conflict of interest.

## Publisher's Note

All claims expressed in this article are solely those of the authors and do not necessarily represent those of their affiliated organizations, or those of the publisher, the editors and the reviewers. Any product that may be evaluated in this article, or claim that may be made by its manufacturer, is not guaranteed or endorsed by the publisher.
